# Integration of Bioinformatics Resources Reveals the Therapeutic Benefits of Gemcitabine and Cell Cycle Intervention in SMAD4-Deleted Pancreatic Ductal Adenocarcinoma

**DOI:** 10.3390/genes10100766

**Published:** 2019-09-28

**Authors:** Yao-Yu Hsieh, Tsang-Pai Liu, Chia-Jung Chou, Hsin-Yi Chen, Kuen-Haur Lee, Pei-Ming Yang

**Affiliations:** 1 Ph.D. Program for Cancer Molecular Biology and Drug Discovery, College of Medical Science and Technology, Taipei Medical University and Academia Sinica, Taipei 11031, Taiwanliutp@mmh.org.tw (T.-P.L.); coll78418@yahoo.com.tw (C.-J.C.); hyichen@tmu.edu.tw (H.-Y.C.); khlee@tmu.edu.tw (K.-H.L.); 2 Division of Hematology and Oncology, Taipei Medical University Shuang Ho Hospital, New Taipei City 23561, Taiwan; 3 Division of Hematology and Oncology, Department of Internal Medicine, School of Medicine, College of Medicine, Taipei Medical University, Taipei 11031, Taiwan; 4 Department of Surgery, Mackay Memorial Hospital, Taipei 10449, Taiwan; 5 Liver Medical Center, Mackay Memorial Hospital, Taipei 10449, Taiwan; 6 Nursing and Management, Mackay Junior College of Medicine, New Taipei City 25245, Taiwan; 7 Department of Medicine, Mackay Medical College, New Taipei City 25245, Taiwan; 8 Graduate Institute of Cancer Biology and Drug Discovery, College of Medical Science and Technology, Taipei Medical University, Taipei 11031, Taiwan; 9 TMU Research Center of Cancer Translational Medicine, Taipei Medical University, Taipei 11031, Taiwan; 10 Cancer Center, Wan Fang Hospital, Taipei Medical University, Taipei 11696, Taiwan

**Keywords:** bioinformatics, cell cycle, in silico, pancreatic ductal adenocarcinoma, SMAD4

## Abstract

Pancreatic ductal adenocarcinoma (PDAC) is the most common and aggressive type of pancreatic cancer. The five-year survival rate of PDAC is very low (less than 8%), which is associated with the late diagnosis, high metastatic potential, and resistance to therapeutic agents. The identification of better prognostic or therapeutic biomarker may have clinical benefits for PDAC treatment. SMAD4, a central mediator of transforming growth factor beta (TGFβ) signaling pathway, is considered a tumor suppressor gene. SMAD4 inactivation is frequently found in PDAC. However, its role in prognosis and therapeutics of PDAC is still unclear. In this study, we applied bioinformatics approaches, and integrated publicly available resources, to investigate the role of SMAD4 gene deletion in PDAC. We found that SMAD4 deletion was associated with poorer disease-free, but not overall, survival in PDAC patients. Cancer hallmark enrichment and pathway analysis suggested that the upregulation of cell cycle-related genes in SMAD4-deleted PDAC. Chemotherapy response profiling of PDAC cell lines and patient-derived organoids revealed that SMAD4-deleted PDAC was sensitive to gemcitabine, the first-line treatment for PDAC, and specific cell cycle-targeting drugs. Taken together, our study provides an insight into the prognostic and therapeutic roles of SMAD4 gene deletion in PDAC, and SMAD4 gene copy numbers may be used as a therapeutic biomarker for PDAC treatment.

## 1. Introduction

The incidence of pancreatic cancer has markedly increased over the past few years [1]. Pancreatic ductal adenocarcinoma (PDAC), the most common and aggressive type of pancreatic cancer, accounts for more than 85% of cases [2]. The five-year survival rate of PDAC is less than 8% [1]. Such high lethality is largely correlated with late diagnosis, high metastatic potential, and resistance to therapeutic agents [3]. Even for patients with early-stage disease, the recurrent rate is still high [4]. Surgical resection remains the only potential curative treatment. However, 80–85% of patients are unresectable. In addition, approximately 50% of patients have metastasis when they are initially diagnosed, leading to the prevention of curative resection [3]. Gemcitabine-based chemotherapy, alone, or in combination with other drugs (such as nab-paclitaxel), and FOLFIRINO (5-fluorouracil, leucovorin, irinotecan, and oxaliplatin) are therapeutic options for advanced pancreatic cancer [4,5]. However, as one of the most chemoresistant cancers, current regimens have not given satisfactory results [6]. Therefore, a better understanding of pancreatic cancer biology will help develop more effective therapeutic strategies.

SMAD4, also known as DPC4 (deleted in pancreatic cancer locus 4), belongs to a member of the SMAD family of signal transducers. It acts as a central mediator for transforming growth factor beta (TGFβ) signaling pathway. TGFβ binds to type II and type I serine/threonine kinase receptors, which mediates phosphorylation and the activation of type I receptor by type II receptor. SMAD2/3 are then phosphorylated by type I receptor and form complexes with SMAD4, which translocate into the nucleus and regulate the transcription of target genes [7,8]. SMAD4 is viewed as a tumor suppressor because it is frequently mutated or deleted in many cancer types, especially in PDAC. Given that TGFβ signaling pathway can be either, tumor-suppressive or tumor-promoting, TGFβ/SMAD4-dependent pathway is considered tumor-suppressive by inducing cell cycle arrest and apoptosis [7,8].

In this study, we investigated the prognostic and therapeutic impacts of SMAD4 deletion in PDAC and identified a potential therapeutic strategy for SMAD4-deleted PDAC, via an integrated bioinformatics analysis, using publicly available resources. We found that PDAC patients with SMAD4 deletion had poorer disease-free survival. Chemosensitivity profiling of PDAC cancer cell lines and patient-derived organoids indicated that SMAD4 deletion sensitized PDAC to gemcitabine and cell cycle-targeting drugs. Mechanistically, SMAD4-deleted cancer cells underwent higher active mitosis, making them more sensitive to gemcitabine and cell cycle intervention.

## 2. Materials and Methods

### 2.1. Cancer Genomics Analysis via The cBioPortal Website

The cBioPortal (http://www.cbioportal.org/) is a website to access, analyze, and visualize the large-scale cancer genomics datasets from TCGA (The Cancer Genome Atlas) or other studies [9,10]. For pan-cancer analysis of SMAD4 genetic alterations, the “TCGA, PanCancer Atlas” datasets were selected. For the further analyses of “Pancreatic Adenocarcinoma (TCGA, PanCancer Atlas)” dataset, 168 PDAC, patients with complete genetic status (mutation, copy number variation, and mRNA expression) were selected with default parameters. SMAD4 gene expression data, associated with mutations and copy number variations in these patients, were retrieved and plotted with Prism software (GraphPad Software, Inc.). Kaplan-Meier survival plots were generated using the cBioPortal and GEPIA (Gene Expression Profiling Interactive Analysis; http://gepia2.cancer-pku.cn/ [11]) databases.

### 2.2. Gene Set Enrichment Analysis (GSEA) and Pathway Construction

Over- and under-expressed genes (Table S1; *p* < 0.01) enriched in SMAD4-deleted PDAC patients were retrieved and subjected to pathway enrichment against 50 cancer hallmarks [12] using the WebGestalt (WEB-based Gene SeT AnaLysis Toolkit; http://www.webgestalt.org/) web-based tool [13]. The gene set enrichment analysis (GSEA) approach was used. Different from the over-representation analysis (ORA) approach that analyzed differentially expressed genes (DEGs), the GSEA approach analyzed all the genes in Table S1 and ranked them by their expression ratio. Alternatively, gene expression profiles of PDAC tumor samples (Table S2) were downloaded from the cBioPortal website and analyzed using the GSEA v3.0 software (http://www.broadinstitute.org/gsea/ [14,15]) for the enrichment of cancer hallmarks [12]. The results were visualized by a Venn diagram using the VENNY 2.1 web tool (https://bioinfogp.cnb.csic.es/tools/venny/). For the enrichment of gemcitabine-sensitive and gemcitabine-resistant genes in SMAD4-null (homozygous deletion [16]) and SMAD4-reexpressed BxPC-3 cells, GSEA v3.0 software was also used. Subsequent KEGG (Kyoto encyclopedia of genes and genomes [17,18,19]) pathway mapping to cell cycle (hsa04110) was also performed using the WebGestalt. Pathway construction was performed using the STRING (Search Tool for the Retrieval of Interacting Genes/Proteins; http://string-db.org/) database [20]. The parameters were set as follows: Organism = homo sapiens; meaning of network edges = molecular action; active interaction source = experiments and databases; minimum required interaction score = high confidence (0.700); max number of interactors to show = none; and network display mode = interactive svg.

### 2.3. Chemosensitivity Profiling in PDAC Cancer Cell Lines and PDAC Patient-Derived Organoids

The relationships between SMAD4 gene alterations and chemosensitivity in PDAC cancer cell lines were obtained from the CTRP (Cancer Therapeutics Response Portal; https://portals.broadinstitute.org/ctrp.v2.1/ [21,22,23]) via an interactive web-based tool, the CellMinerCDB (https://discover.nci.nih.gov/cellminercdb/ [24]). The CTRP database contains small-molecule sensitivity profiles in various cancer cell lines with annotated genetic and cellular features. It contains the in vitro chemosensitivity profiles from 860 cancer cell lines treated with 481 compounds for 72 hours (8 concentrations per drug) [21,22,23]. The CellMinerCDB is an interactive web-based portal for querying and visualizing related genomics and pharmacological data from large-scale cancer cell lines [24]. The chemosensitivity data in PDAC patient-derived organoids with different SMAD4 gene status can be obtained from a previous study [25]. The raw data used to generate Figure 4c were shown in Table S5.

### 2.4. Materials

Roswell Park Memorial Institute (RPMI)-1640 and Dulbecco’s Modified Eagle Medium (DMEM) medium, glucose, L-glutamine, sodium pyruvate, and antibiotic-antimycotic solution (amphotericin B, penicillin, streptomycin) were purchased from Life Technologies. Fetal bovine serum (FBS) was purchased from Biological Industries. Gemcitabine, dimethyl sulfoxide (DMSO), and 3-(4,5-dimethylthiazol-2-yl)-2,5-diphenyl tetrazolium bromide (MTT) were purchased from Sigma. The 2X KAPA SYBR FAST qPCR Master Mix was purchased from Kapa Biosystems. The iScript cDNA Synthesis Kit was purchased from Bio-Rad.

### 2.5. Cell Culture and MTT Cell Viability Assay

AsPC-1, BxPC-3, HPAC, and PANC-1 human pancreatic cancer cells were purchased from the Bioresource Collection and Research Center (BCRC), Food Industry Research and Development Institute (Hsinchu, Taiwan). AsPC-1 and BxPC-3 were cultured in RPMI-1640 medium and HPAC and PANC-1 cells were cultured in DMEM medium. These media were supplemented with 10% FBS, 4.5 g/mL glucose, 2 mM L-glutamine, 1 mM sodium pyruvate, 1% antibiotic-antimycotic. The cells were passaged every 2 or 3 days and cultured at 37 °C and 5% CO_2_ in a humidified incubator. Cell viability after drug treatment was determined by an MTT assay. Briefly, cells (5 × 10^3^ cells/well) were plated in 96-well plates and treated with drugs for 72 h. Then, 0.5 mg/mL MTT was added 4 h before cell harvest. Blue MTT formazan precipitates were dissolved in 200 μL DMSO. The absorbance at 550 nm was measured on a multi-well plate reader.

### 2.6. Real-Time Quantitative Polymerase Chain Reaction (qPCR) and Western Blot Analysis

The total RNA was purified by GENEzol TriRNA Pure Kit. The first-strand cDNA was synthesized using the iScript cDNA Synthesis Kit and then PCR was performed using the 2X KAPA SYBR FAST qPCR Master Mix using the following primer pairs. SMAD4: forward, 5′-TGGAATGTAAAGGTGAAG GTGA-3′ and reverse, 5′-GACACTGACGCAAATCAAAGAC-3′; β-Actin: forward, 5′-GTTGCTATCC AGGCTGTGCT-3′ and reverse, 5′-AGGGCATACCCCTCGTAGAT-3′. Each assay was performed on a LightCycler 96 System (Roche). The fold-changes in expression were derived using the comparative CT method calculated by LightCycler 96 Software v1.1.0.1320 (Roche).

### 2.7. Statistical Analysis

Statistical analysis was performed by built-in programs in each database or software used in this study. For plots generated by the Prism software, a two-tailed unpaired Student′s *t* test was performed. A *p* value of < 0.05 was considered significant.

## 3. Results

### 3.1. SMAD4 Gene Deletion Predicts A Poorer Disease-Free Survival in PDAC Patients

**Figure 1:**
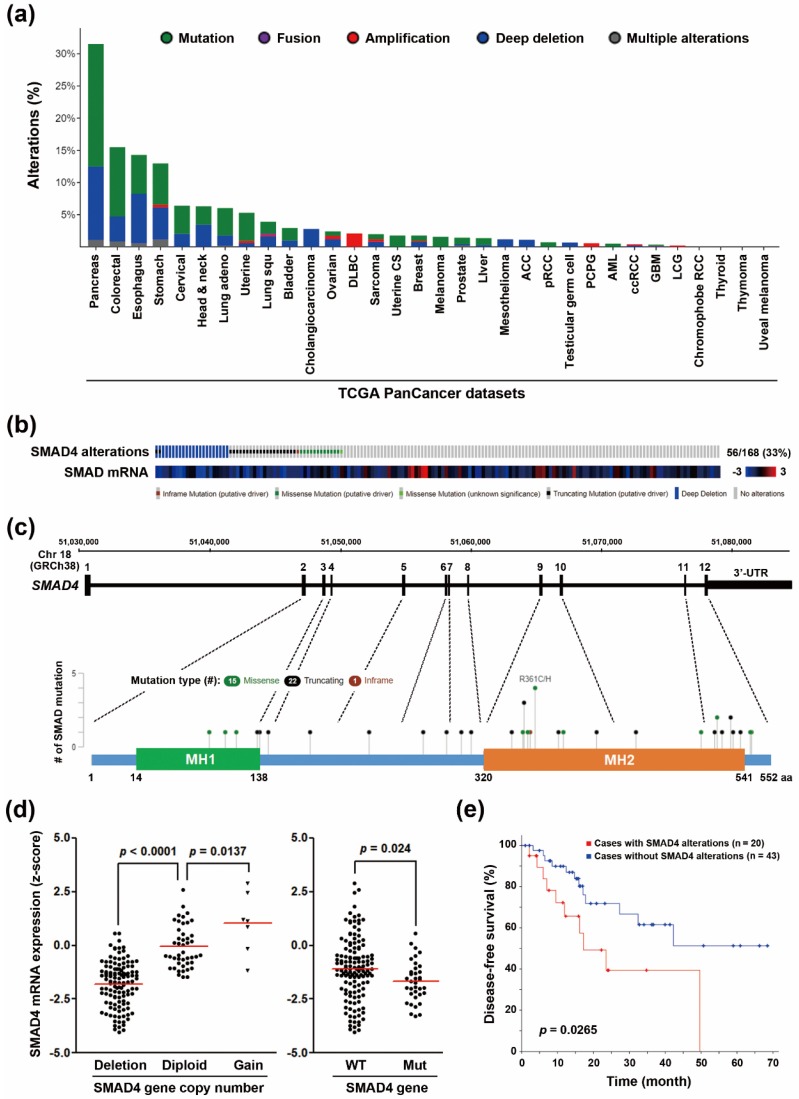
Copy number amplification of SMAD4 gene in pancreatic ductal adenocarcinoma (PDAC). (**a**) SMAD4 gene was analyzed for gene alterations (mutation status and copy number variation) in various cancer types using “TCGA, PanCancer Atlas” data in the cBioPortal cancer genomics database; (**b**) A bar code plot for the comparison of SMAD4 gene alterations and mRNA expression in PDAC; (**c**) A cartoon diagram for the gene and protein structures of SMAD4. MH1 (the Mad homology 1) region is sequence-specific DNA binding domain. MH2 region is responsible for heteromerization and transactivation. This figure was adapted from the image obtained from the cBioPortal website (http://www.cbioportal.org/); (**d**) A scatter plot for the comparison of SMAD4 copy number amplification and mRNA expression in PDAC. WT, wildtype. Diploid, two alleles present; Gain, low-level gene amplification event; Mut, mutation; (**e**) The impact of SMAD4 gene alterations on disease-free survival of PDAC patients.

SMAD4 inactivation is frequently found in PDAC [7,26]. Indeed, we mined “TCGA, PanCancer Atlas” data via the cBioPortal website (http://www.cbioportal.org/) [9,10] for the genetic alterations of SMAD4 gene, and found that 33% of PDAC patients harbored SMAD4 gene mutation and copy number loss (Figure 1a,b). This pan-cancer analysis also indicated that SMAD4 gene alterations occurred most frequently in PDAC, compared with other cancer types (Figure 1a). Among 168 PDAC patients, 36 patients carried mutations and 22 patients displayed gene deletions, 2 of which simultaneously harbored SMAD4 gene mutations and deletions (Figure 1b). From the diagram of SMAD4 gene and the encoded protein, mutations occurred more frequently in the MAD homology 2 (MH2) domain that is responsible for heteromerization and transactivation (Figure 1c). SMAD4 gene mutation and copy number loss were positively correlated with the reduction in SMAD4 mRNA levels (Figure 1d). PDAC patients, with SMAD4 gene alterations, had similar overall survival rate (Figure S1a). However, they had a shorter disease-free survival (Figure 1e). In addition, the prognostic impact of SMAD4 gene alterations on disease-free survival was largely attributed to the loss of gene copy numbers (Figure S1b,c). Therefore, SMAD4 gene deletion may predict poorer disease-free survival in PDAC patients. To investigate whether the SMAD4-deleted tumors were biased towards a more aggressive subtype, we ranked SMAD4 gene deletions and mutations by the histological grades and tumor stages. As shown in Figure S2, SMAD4-deleted and SMAD4-mutated tumors distributed similarly. Therefore, the differential prognostic impacts of SMAD4 gene deletion and mutation on PDAC patients’ disease-free survivals are unrelated to their grades/stages.

### 3.2. Upregulation Of Cell Cycle-Related Genes in SMAD4-Deleted PDAC

**Figure 2:**
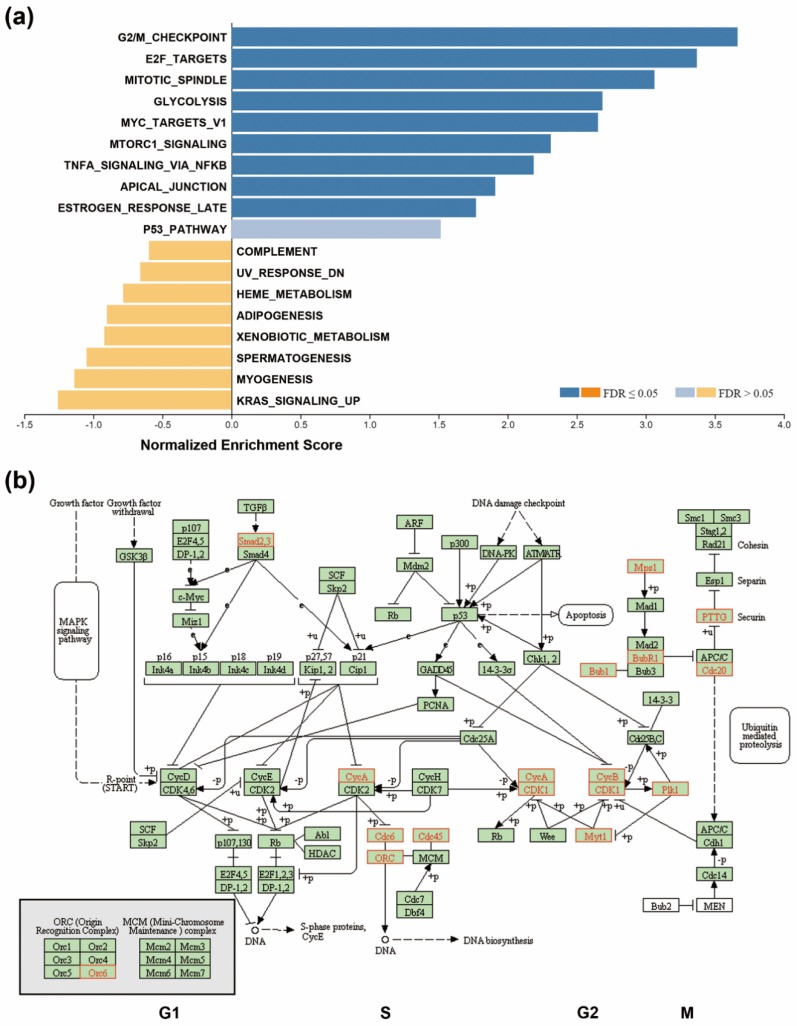
Upregulation of cell cycle-related genes in SMAD4-deleted PDAC. (**a**) Gene set enrichment analysis (GSEA) for the over- and under-expressed genes in SMAD4-deleted PDAC patients was performed using the WebGestalt online tool; (**b**) Kyoto Encyclopedia of Genes and Genomes (KEGG) cell cycle pathway mapping was performed using the WebGestalt online tool. The official gene symbols for the mapped genes were annotated and highlighted in red (over-expressed) and blue (under-expressed). No under-expressed genes were enriched in this pathway.

To investigate the changes in gene expression profiles in SMAD4-deleted PDAC, 22 SMAD4-deleted PDAC patients were selected from “TCGA, PanCancer Atlas” dataset, and then the most significantly over- and under-expressed genes enriched in these patients (versus the other 146 patients without SMAD4 deletion) were obtained using the “enrichments” tool in the cBioPortal website. The full gene list was shown in Table S1. Cancer hallmark enrichment of these genes were performed using the WebGestalt database (http://www.webgestalt.org/) [13]. As shown in Figure 2a, the hallmarks, enriched in downregulated genes, were not statistically significant (FDR > 0.05). However, the hallmarks related to cell cycle progression (such as G2/M checkpoint, E2F targets, and mitotic spindle) were enriched in the upregulated genes. The cell cycle pathway for the upregulated genes was further constructed using the KEGG cell cycle pathway map (hsa04110) [17,18,19]. As shown in Figure 2b, the major network consisted of many genes playing essential roles during the G2 phase and mitosis. Specifically, cyclin-dependent kinase 1 (CDK1) and its binding partners, cyclin A (CycA) and B (CycB) were upregulated. CDK1, together with polo-like kinase 1 (PLK1), were required for mitotic entry [27]. These analyses suggest that active cell cycle progression is a major phenotype in SMAD4-deleted PDAC.

To confirm the above analyses and compare the differential effects of SMAD4 gene deletion and mutation, the gene expression profiles of PDAC patients (patients’ IDs were shown in Table S2) were downloaded from the cBioPortal website and analyzed using the GSEA software. We compared the three groups through pairwise method (SMAD4-deletion/DEL, *n* = 20; SMAD4-mutation/MUT, *n* = 34; and SMAD4-wildtype/WT, *n* = 112). The original results were shown in Table S3 and a Venn diagram showed the overlapped cancer hallmarks between DEL versus WT and MUT versus WT comparisons (Figure S3a). Despite not being statistically significant, cell cycle-related hallmarks (such as G2/M_CHECKPOINT, E2F_TARGETS, and DNA_REPAIR) were upregulated in SMAD4-DEL, but not SMAD4-MUT group when compared to SMAD4-WT group (Figure S3a). However, a statistically significant enrichment (*p* < 0.05) for these three hallmarks was found when SMAD4-DEL group was compared with SMAD4-MUT group (Table S3 and the enrichment plots were shown in Figure S3b). Therefore, these analyses further support the theory that SMAD4 gene deletion, but not mutation, differentially affects the expression of cell cycle-related genes.

### 3.3. SMAD4 Gene Deletion is Associated With an Increased Sensitivity To Cell Cycle-Targeting Drugs in PDAC Cell Lines

**Figure 3:**
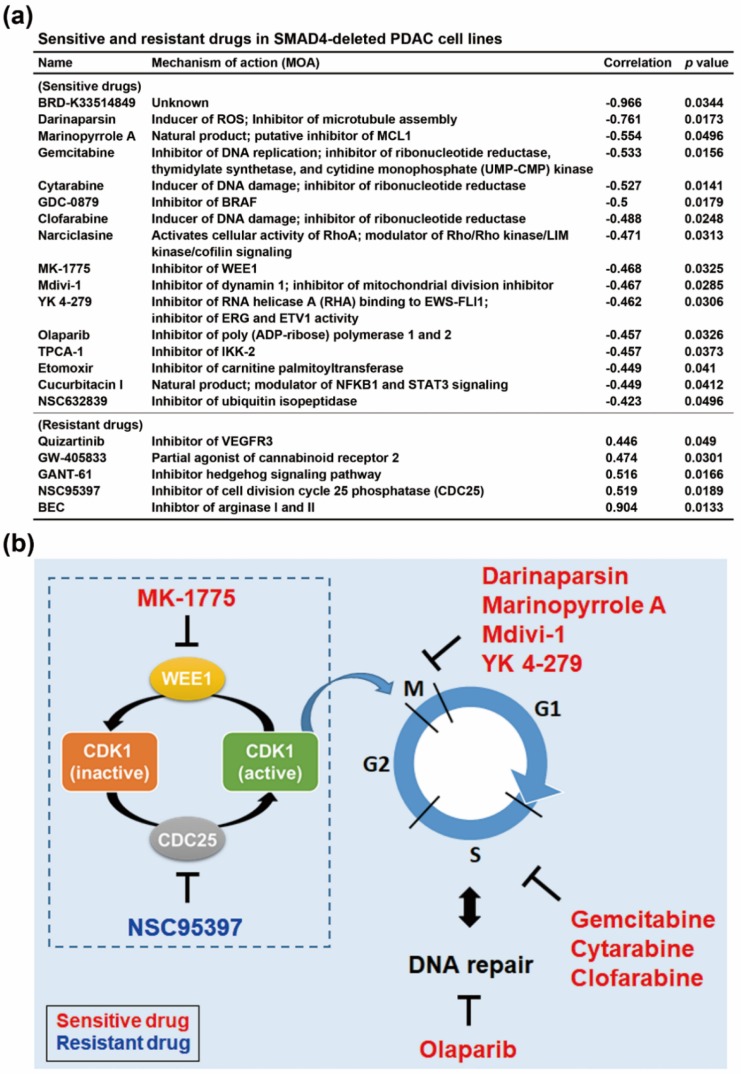
Sensitive and resistant drugs in SMAD4-deleted PDAC cell lines. (**a**) Chemosensitivity profiles, correlated with SMAD4 copy numbers in PDAC cell lines, were obtained from the CTRP database via an online tool, the CellMinerCDB; (**b**) The effect of drugs on cell cycle progression.

PDAC usually responds poorly to most chemotherapeutic agents, and furthering our understanding of the relationship between individual genetic profiles and chemosensitivity might improve the efficacy of chemotherapy. Therefore, we searched a potential therapeutic strategy to treat SMAD4-deleted PDAC by mining the CTRP (https://portals.broadinstitute.org/ctrp.v2.1/) database [21,22,23] via a web-based tool, CellMinerCDB (https://discover.nci.nih.gov/cellminercdb/) [24]. The CTRP database consists of basal gene expression profiles of cancer cell lines correlated with drug sensitivity [21,22,23]. The drugs positively (sensitive drugs) and negatively (resistant drugs) correlated with SMAD4 gene copy numbers were shown in Figure 3a and Table S4. We found that the predicted sensitive drugs for SMAD4-deleted PDAC cells mainly targeted cell cycle progression (Figure 3b), which was consistent with our finding that upregulation of cell cycle-related genes in SMAD4-deleted PDAC (Figure 2). Among these sensitive drugs, gemcitabine, cytarabine, and clofarabine inhibited ribonucleotide reductase, leading to replication stress [28]. Olaparib, a poly ADP-Ribose polymerase 1 (PARP1) inhibitor, inhibited DNA repair [29]. The WEE1 inhibitor, MK-1775, induced premature CDK1 activation and abrogated G2 DNA damage checkpoint [30]. Opposite to WEE1, CDC25C phosphorylates and activated CDK1. Interestingly, CDC25C inhibitor, NSC95397, was predicted as a resistant drug for SMAD4-deleted PDAC cells. Thus, activation of CDK1 may correlated with increased drug sensitivity in SMAD4-deleted cells. We also found that several drugs having the potential to disrupt mitosis, including darinaparsin (an inhibitor of microtubule assembly) [31], marinopyrrole A (an inhibitor of MCL1 that is degraded during mitotic arrest) [32,33], mdivi-1 (induction of multipolar acentrosomal mitotic spindles) [34], and YK 4-279 (inhibition of kinetochore microtubule formation) [35]. Taken together, we hypothesized that SMAD4-deleted PDAC cells undergo active mitosis, thus exhibiting higher sensitivity to cell cycle-targeting agents.

### 3.4. Increased Gemcitabine Sensitivity in Both SMAD4-Deleted PDAC Cell Lines and PDAC Patients-Derived Organoids

**Figure 4:**
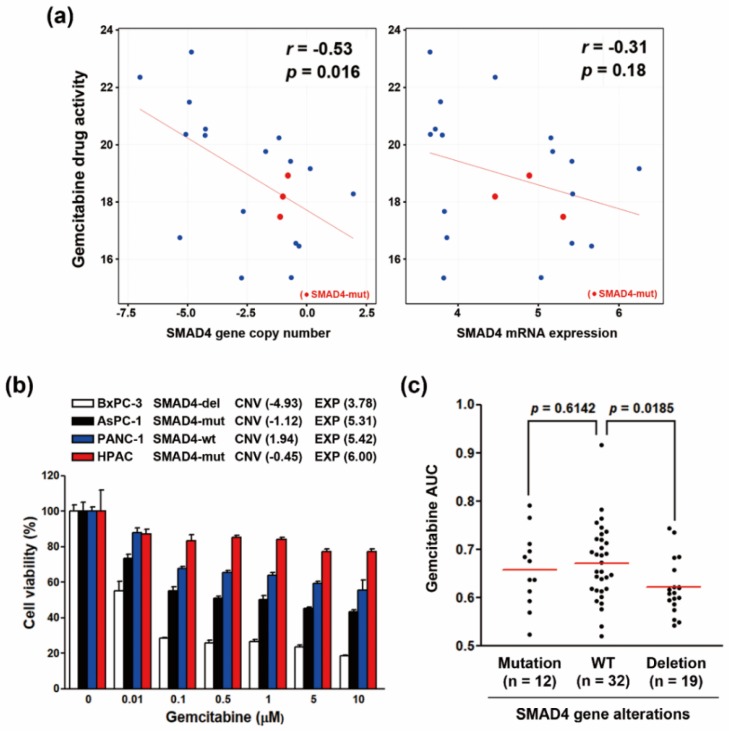
SMAD4 deletion correlated with an increase in gemcitabine sensitivity in PDAC. (**a**) The correlations between SMAD4 gene copy numbers (left part)/mRNA expression (right part) and gemcitabine drug activity were obtained from the CTRP database via an online tool, the CellMinerCDB; (**b**) PDAC cell lines were treated with gemcitabine for 72 h, and then cell viability was determined by an MTT assay. The SMAD4 mutation status was obtained from the CCLE database (missence mutation and frameshift insertion in AsPC-1, and HPAC cells, respectively). BxPC-3 cells harbored a homozygous deletion of SMAD4 gene [16]. The values for SMAD4 copy number variation (CNV) and expression (EXP) were obtained from the CellMinerCDB database (CTRP-Broad-MIT data); (**c**) Chemosensitivity profiles for PDAC patient-derived organoids were obtained from a previous study. Values shown are the area under the curve (AUC) for each drug. Higher or lower AUC (area under the curve) indicated less or more responsive to each drug, respectively.

Gemcitabine is one of the standard chemotherapy for PDAC [4], which was predicted as a sensitive drug for PDAC cells with low SMAD4 gene copy number (Figure 3a). Therefore, we further investigated in detail the relationship between gemcitabine sensitivity and SMAD4 gene alterations in PDAC. Unlike SMAD4 gene deletion (Figure 4a, left part), SMAD4 mRNA expression was not significantly correlated with the sensitivity of PDAC cell lines to gemcitabine (Figure 4a, right part). Because only three cell lines harbored a SMAD4 gene mutation (the red dots in Figure 4a), we could not conclude whether there is a correlation between gemcitabine sensitivity and SMAD4 gene mutation. To understand this further, four PDAC cell lines with different SMAD4 genetic status were treated with gemcitabine, and cell viability was measured by MTT assay. As shown in Figure 4b, BxPC-3 cell line with the lowest SMAD4 copy number and mRNA expression was the most sensitive one to gemcitabine. To confirm the relative SMAD4 expression in these four PDAC cell lines, real-time quantitative polymerase chain reaction (qPCR) was performed. As shown in Figure S4, the SMAD4 mRNA expression was well-correlated with the database-retrieved values (Figure 4b).

Controversially, a previous study using a genetically-defined pancreatic cancer cell panel, which was used to test their responses to chemotherapeutic drugs, found that SMAD4-inactivated cancer cell lines are more sensitive to cisplatin and irinotecan, but modestly (not statically significant) less sensitive to gemcitabine [36]. To further support our findings and investigate the clinical implication, the drug response data in PDAC patient-derived organoids, with different SMAD4 genetic alterations, were obtained from a previous study (Table S5) [25]. Consistently, SMAD4-deleted, but not SMAD4-mutated, organoids were more sensitive to gemcitabine than SMAD4-wildtype (WT) ones (Figure 4c). The above observations were also compared with other clinical drugs for treating PDAC, including paclitaxel, 5-fluorouracil (5-FU), and SN-38 (the active metabolite of irinotecan). Although, there was a trend that PDAC cells and PDAC patient-derived organoids with lower SMAD4 gene copy numbers were sensitive to these drugs, their correlations were not statistically significant except for SN-38-treated organoids (Figure S5). Therefore, the SMAD4 gene copy number could be used as a therapeutic biomarker for treating PDAC with gemcitabine.

### 3.5. The Association Between CDK1 and Genes Correlated with Gemcitabine Sensitivity in SMAD4-Deleted PDAC Patient-Derived Organoids

**Figure 5:**
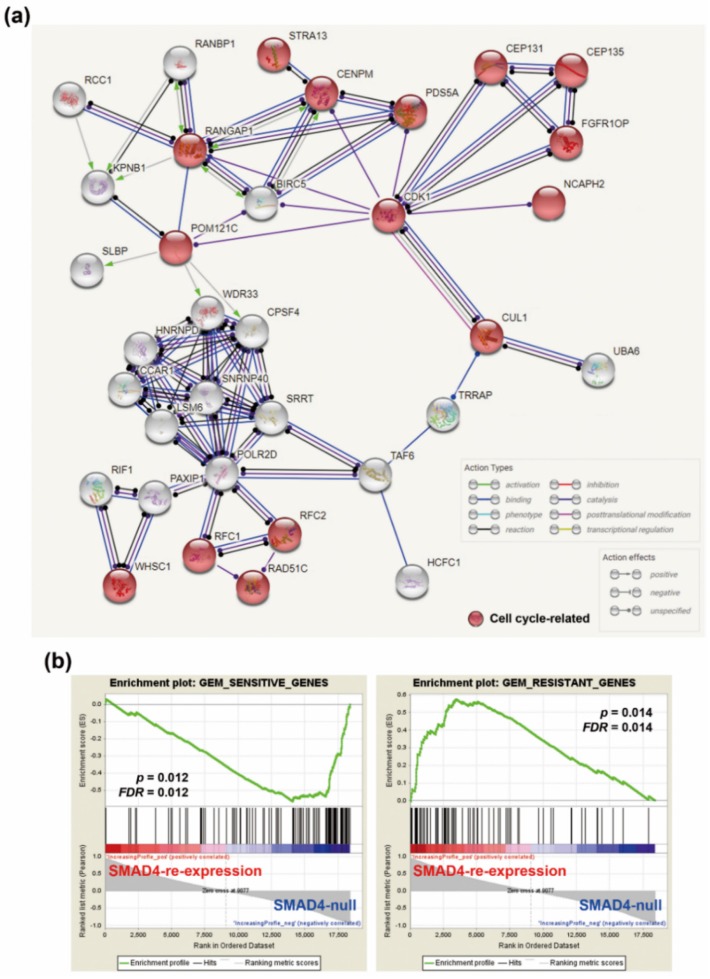
Gemcitabine-sensitive genes were closely associated with CDK1 and SMAD4 gene deletion in PDAC. (**a**) The network between CDK1 and gemcitabine-sensitive genes was constructed using the STRING database; (**b**) GSEA was performed to analyze the correlation between gemcitabine-sensitive/resistant genes and SMAD4 gene expression.

To further investigate whether the increased gemcitabine sensitivity in SMAD4-deleted PDAC patient-derived organoids was also attributed to the upregulation of cell cycle-related genes, the genes positively and negatively correlated with gemcitabine sensitivity in PDAC patient-derived organoids were obtained from the previous study [25]. The gene list was shown in Table S6. Their connections to CDK1 were constructed using the STRING (https://string-db.org/) [20]. We found that gemcitabine-sensitive genes, but not gemcitabine-resistant genes, can form a network to CDK1 (Figure 5a, Figures S6 and S7), suggesting that upregulated of CDK1-related genes was associated with gemcitabine sensitivity in SMAD4-deleted PDAC patient-derived organoids. To further support this finding, the expression of gemcitabine-sensitive and gemcitabine-resistant genes (Table S6) in a gemcitabine-sensitive, SMAD4-null BxPC-3 cell line and its SMAD4-inducible clone (GSE70940 [37]) was analyzed using the GSEA software. Consistently, gemcitabine-sensitive genes were enriched in SMAD4-null BxPC-3 cells, whereas gemcitabine-resistant genes were enriched in SMAD4-re-expressed BxPC-3 cells (Figure 5b). Therefore, the upregulation of cell cycle-related genes contributes to an increase in gemcitabine sensitivity in SMAD4-deleted PDAC.

## 4. Discussion

TGFβ signaling pathways play both pro-tumorigenic and tumor-suppressive roles in pancreatic cancer, depending on the stage of the tumor stage the microenvironment. Alterations of TGFβ signaling components, such as SMAD4 and TGFβ type II receptor (TGFBRII), are common during pancreatic tumorigenesis [26,38]. At early stages of tumorigenesis, TGFβ signaling pathway have tumor-suppressive activity because TGFβ functions as an anti-mitogen, that blocks cell cycle progression at G1 phase, preventing RB hyper-phosphorylation and S phase entry [39]. It is undoubtedly that SMAD4 loss causes the insensitivity of cancer cells to the anti-mitogenic activity of TGFβ, and then abrogating RB-mediated E2F1 suppression. Indeed, GSEA indicated that the cancer hallmark “E2F_TARGETS” was enriched in SMAD4-deleted PDAC (Figure 2a), accounting for the upregulation of cell cycle-related gene expression. However, in late stage tumorigenesis, TGFβ promotes cancer invasion, angiogenesis, and metastasis as it acts as a major inducer of epithelial-to-mesenchymal transition (EMT) [26,38]. Although, TGFβ-induced EMT is considered a pro-tumorigenic effect, it becomes lethal in TGFβ-sensitive (SMAD4-wildtype) pancreatic cancer by converting TGFβ-induced Sox4 from an enforcer of tumorigenesis into a promoter of apoptosis [40]. Therefore, the accumulation of SMAD4 mutations and deletions in pancreatic cancer obstructs the tumor-suppressive element, but enhances the pro-tumorigenic effect of TGFβ.

Drug sensitivity profiling in PDAC cell lines suggested that active CDK1 might be an indicator for increased gemcitabine sensitivity (Figure 3). WEE1 and CHK1 are two important kinases that inactivate CDK1 [41]. It has been found that the pharmacological inhibition of WEE1 and CHK1 sensitizes pancreatic cancers to gemcitabine [42,43]. It is possible that active CDK1 disables DNA repair, resulting in persistent genomic damages [42,43]. This hypothesis can be supported by a chemical screening using isogenic BxPC-3 cell lines with, or without, SMAD4 gene deletion, in which a small-molecule UA62784 is identified as a selectively cytotoxic agent to kill SMAD4-deficient pancreatic and colon cancer cells [44]. Mechanistically, UA62784 preferentially activates CDK1 and induces cell cycle arrest and apoptosis in cancer cells with deficient SMAD4 [44,45]. Our results suggest that cell cycle-dependent drug intervention is an alternative option for treating PDAC.

Our results indicate that SMAD4 gene deletion, but not mutation, predicted poorer disease-free survival in PDAC patients (Figure S1b,c). Although, SMAD4 mRNA downregulation were associated with both mutations and deletions (Figure 1d), SMAD4 mRNA expression was not associated with the overall, and disease-free survivals of PDAC patients (Figure S8). Such discordance suggests that SMAD4 copy number variation is a better prognostic biomarker. In addition, SMAD4 loss may have resulted from genetic and/or epigenetic mechanisms. For example, microRNAs 301a-3p, 421, 483-3p have been shown to downregulate SMAD4 [46,47,48]. Furthermore, our results also support the principle that clearly defined SMAD4 loss (including mutation and deletion) is required in the future when investigating its role in patients’ prognostic impacts.

We also found that SMAD4 alterations (especially deletion) did not predict the overall survival in PDAC patients (Figure 1e and Figure S1). It is possible that small number of cases did not reach the true predictive power. However, controversial results for the prognostic value of SMAD4 are also found in previous studies. For example, Oshima M et al. [49] shows that SMAD4 loss is associated with poor overall and disease-free survivals in resectable pancreatic cancer. Winter JM et al. [50] found that SMAD loss is neither associated with recurrent pattern, nor associated with early death in resectable pancreatic cancer. Ormanns S et al. [51] found that SMAD4 loss has no impact on overall survival, but has a prolonged progression-free survival in gemcitabine-treated advanced pancreatic cancer. Such contradiction warrants further investigation.

Two-dimensional (2D) monolayer cancer cell culture has been an important tool for basic cancer research and drug discovery. However, the gap between in vitro and in vivo studies still exists, leading to their failure in clinical settings. Therefore, more clinically relevant models are needed. Three-dimensional (3D) patient-derived organoids can recapitulate the in vivo tumor growth and maintain the original genetic and histological features [52], providing a better model in drug screening to support results from conventional 2D monolayer. In this study, both SMAD4-deleted pancreatic cancer cell lines and patient-derived organoids exhibited higher sensitivity toward gemcitabine (Figure 4a,c), supporting the use of SMAD4 gene copy number variation as a therapeutic biomarker for treating pancreatic cancer patients.

Tumor analysis is usually limited by the initial biopsy or surgery. Rapid autopsy is a unique methodology, which collected tissue samples from consented cancer patients on an urgent basis (usually in hours). Rapid autopsy enables multiple tumor loci to be found (especially the metastatic sites) and normal control tissues from the same cancer patient, which is not easily done by traditional biopsy, thereby facilitating to decipher the tumor heterogeneity and the molecular mechanism for tumor growth and spread [53]. Through rapid autopsies from pancreatic cancer patients, Yachida S et al. [54] and Iacobuzio-Donahue CA et al. [55] found that SMAD4 is inactivated during subclonal evolution and metastatic progression, but is not associated with locally destructive tumors. In addition, Herman JM et al. [56] found that SMAD4 loss correlates with higher rates of local and distant failure in PDAC receiving adjuvant chemo-radiation therapy. Significant future potential exists in the development of tumor organoids or patient-derived xenografts, from rapid autopsies, will provide more comprehensive models for high-throughput drug screening and functional validation. 

In conclusion, this study integrates publicly available resources to investigate the prognostic and therapeutic impacts of SMAD4 deletion in PDAC. Our results suggest that the loss of SMAD4 gene copy number predicts poor disease-free, but not overall, survival in PDAC patients. In addition, SMAD4-deleted PDAC cells are sensitive to gemcitabine and other cell cycle-targeting agents, due to the upregulation of cell cycle-related genes. Our study provides an insight into the prognostic and therapeutic roles of SMAD4 gene deletion in PDAC.

## Supplementary Materials

The following are available online at https://www.mdpi.com/2073-4425/10/10/766/s1, Figure S1: The prognostic impacts of SMAD4 gene alterations on PDAC patient survivals, Figure S2: The distribution of SMAD4 gene deletions and mutations in PDAC patients’ grades/stages, Figure S3: GSEA results for pariwise comparisons of SMAD4-DEL, SMAD4-MUT, and SMAD4-WT, Figure S4: The SMAD4 mRNA expression in PDAC cell lines. Figure S5: The relationship between drug sensitivity and SMAD4 gene copy number variation in PDAC cell lines and patient-derived organoids, Figure S6: Pathway construction for the relationship between CDK1 and gemcitabine-sensitive genes, Figure S7: Pathway construction for the relationship between CDK1 and gemcitabine-resistant genes, Figure S8: The prognostic impacts of SMAD4 mRNA expression on PDAC patient survivals, Table S1: Over- and under-expressed genes enriched in SMAD4-deleted PDAC patients, Table S2: PDAC patients samples for GSEA, Table S3: Pairwise GSEA for SMAD4-deletion (DEL), SMAD4-mutation (MUT), and SMAD4-wildtype (WT), Table S4: The SMAD4 gene copy number and gemcitabine drug activity of PDAC cell lines, Table S5: Chemosensitivity of PDAC patient-derived organoids, Table S6: Genes correlated with gemcitabine sensitivity in PDAC patient-derived organoids.
