# Supplementary material for: Integration of Bioinformatics Resources Reveals the Therapeutic Benefits of Gemcitabine and Cell Cycle Intervention in SMAD4-Deleted Pancreatic Ductal Adenocarcinoma
Source: Genes (Basel). 2019 Sep 28;10(10):766. doi: 10.3390/genes10100766 (PMC6827004; doi:10.3390/genes10100766)
Supplement: Supplementary file 1 [file genes-10-00766-s001.zip › Table S4.docx]

**Table S4. The SMAD4 gene copy number and gemcitabine drug activity of PDAC cell lines.** The original values for Figure 3a, which were obtained from the CellMinerCDB database (CTRP-Broad-MIT data).

| **Cell line** | **SMAD4 copy number** | **Gemcitabine drug activity** |
| --- | --- | --- |
| AsPC-1 | -1.1191 | 17.48 |
| BxPC-3 | -4.9319 | 21.4842 |
| Capan-1 | -0.7999 | 18.916 |
| Capan-2 | -0.6528 | 15.356 |
| CFPAC-1 | -5.0732 | 20.3508 |
| HPAF-II | -0.3286 | 16.453 |
| Hs 766T | -5.333 | 16.75 |
| KP-3 | -0.4598 | 16.559 |
| KP4 | -4.8463 | 23.23 |
| MIA PaCa-2 | 0.141 | 19.159 |
| Panc 03.27 | -7.0027 | 22.3458 |
| Panc 08.13 | -1.0029 | 18.175 |
| PANC-1 | 1.942 | 18.274 |
| Panc 10.05 | -0.6878 | 19.413 |
| PA-TU-8988S | -2.6699 | 17.67 |
| PA-TU-8988T | -2.7437 | 15.343 |
| PSN1 | -4.2529 | 20.5382 |
| SU.86.86 | -1.7306 | 19.7604 |
| SUIT-2 | -4.2688 | 20.3235 |
| SW 1990 | -1.1694 | 20.2302 |
