# Supplementary material for: Integration of Bioinformatics Resources Reveals the Therapeutic Benefits of Gemcitabine and Cell Cycle Intervention in SMAD4-Deleted Pancreatic Ductal Adenocarcinoma
Source: Genes (Basel). 2019 Sep 28;10(10):766. doi: 10.3390/genes10100766 (PMC6827004; doi:10.3390/genes10100766)
Supplement: Supplementary file 1 [file genes-10-00766-s001.zip › Table S5.docx]

**Table S5. Chemosensitivity of PDAC patient-derived organoids.** Values shown are the area under the curve (AUC) for each drug. Higher or lower AUC indicated less or more responsive to each drug, respectively. *Data source: Cancer Discovery. 2018;8:1112-1129.*

| **Organoid ID** | **SMAD4 status** | **Gemcitabine** | **Paclitaxel** | **5-FU** | **SN-38** |
| --- | --- | --- | --- | --- | --- |
| hF31 | WT | 0.619 | 0.561 | 0.760 | 0.541 |
| hF39 | WT | 0.756 | 0.699 | 0.807 | 0.723 |
| hF43 | WT | 0.614 | 0.618 | 0.834 | 0.544 |
| hF45 | WT | 0.654 | 0.681 | 0.855 | 0.715 |
| hF70 | WT | 0.917 | 0.759 | 0.972 | 0.880 |
| hF72 | WT | 0.619 | 0.558 | 0.852 | 0.550 |
| hF78 | WT | 0.733 | 0.687 | 0.859 | 0.680 |
| hF81 | WT | 0.783 | 0.634 | 0.898 | 0.610 |
| hF82 | WT | 0.688 | 0.707 | 0.889 | 0.674 |
| hM19A | WT | 0.746 | 0.674 | 0.842 | 0.568 |
| hM19B | WT | 0.738 | 0.696 | 0.915 | 0.600 |
| hM19C | WT | 0.673 | 0.630 | 0.664 | 0.590 |
| hM19D | WT | 0.695 | 0.618 | 0.603 | 0.579 |
| hM1A | WT | 0.593 | 0.550 | 0.642 | 0.577 |
| hM1E | WT | 0.690 | 0.714 | 0.687 | 0.639 |
| hM1F | WT | 0.720 | 0.698 | 0.838 | 0.696 |
| hT1 | WT | 0.589 | 0.566 | 0.670 | 0.582 |
| hT102 | WT | 0.541 | 0.560 | 0.850 | 0.550 |
| hT103 | WT | 0.645 | 0.633 | 0.941 | 0.680 |
| hT106 | WT | 0.704 | 0.679 | 0.956 | 0.735 |
| hT108 | WT | 0.520 | 0.547 | 0.747 | 0.496 |
| hT117 | WT | 0.640 | 0.587 | 0.596 | 0.554 |
| hT133 | WT | 0.713 | 0.658 | 0.881 | 0.640 |
| hT134 | WT | 0.602 | 0.577 | 0.818 | 0.564 |
| hT25 | WT | 0.648 | 0.590 | 0.902 | 0.638 |
| hT3 | WT | 0.654 | 0.514 | 0.662 | 0.566 |
| hT44 | WT | 0.577 | 0.582 | N/A | 0.553 |
| hT48 | WT | 0.616 | 0.568 | 0.775 | 0.566 |
| hT64 | WT | 0.643 | 0.558 | 0.710 | 0.589 |
| hT83 | WT | 0.692 | 0.636 | 0.840 | 0.636 |
| hT89 | WT | 0.765 | 0.731 | 0.926 | 0.698 |
| hT91 | WT | 0.722 | 0.572 | 0.745 | 0.564 |
| hF24 | Deletion | 0.610 | 0.742 | 0.671 | 0.549 |
| hF32 | Deletion | 0.550 | 0.577 | 0.911 | 0.617 |
| hF44 | Deletion | 0.608 | 0.561 | 0.718 | 0.559 |
| hF54 | Deletion | 0.587 | 0.591 | 0.728 | 0.565 |
| hF57 | Deletion | 0.683 | 0.584 | 0.745 | 0.597 |
| hF68 | Deletion | 0.601 | 0.666 | 0.761 | 0.622 |
| hF71 | Deletion | 0.575 | 0.669 | 0.750 | 0.503 |
| hF74 | Deletion | 0.685 | 0.600 | 0.782 | 0.589 |
| hF77 | Deletion | 0.744 | 0.597 | 0.764 | 0.659 |
| hM8 | Deletion | 0.658 | 0.626 | 0.855 | 0.641 |
| hT101 | Deletion | 0.622 | 0.575 | 0.653 | 0.545 |
| hT60 | Deletion | 0.618 | 0.596 | 0.804 | 0.537 |
| hT82 | Deletion | 0.598 | 0.735 | 0.643 | 0.594 |
| hT85 | Deletion | 0.543 | 0.577 | 0.618 | 0.530 |
| hF2 | Deletion | 0.554 | 0.713 | 0.783 | 0.500 |
| hF3 | Deletion | 0.620 | 0.544 | 0.719 | 0.564 |
| hT105 | Deletion | 0.622 | 0.521 | 0.803 | 0.572 |
| hT127 | Deletion | 0.736 | 0.680 | N/A | 0.580 |
| hT98 | Deletion | 0.595 | 0.542 | 0.862 | 0.528 |
| hT125 | Mutation | 0.569 | 0.610 | 0.845 | 0.581 |
| hM17D | Mutation | 0.638 | 0.654 | 0.828 | 0.685 |
| hT123 | Mutation | 0.593 | 0.600 | 0.859 | 0.569 |
| hF28 | Mutation | 0.685 | 0.663 | 0.862 | 0.651 |
| hT81 | Mutation | 0.767 | 0.723 | 0.784 | 0.636 |
| hT87 | Mutation | 0.613 | 0.665 | 0.790 | 0.564 |
| hT58 | Mutation | 0.791 | 0.829 | 0.884 | 0.816 |
| hF23 | Mutation | 0.524 | 0.487 | 0.782 | 0.548 |
| hT93 | Mutation | 0.696 | 0.562 | 0.867 | 0.610 |
| hF50 | Mutation | 0.638 | 0.641 | 0.723 | 0.582 |
| hF27 | Mutation | 0.677 | 0.637 | 0.839 | 0.568 |
| hT30 | Mutation | 0.712 | 0.659 | 0.885 | 0.597 |
