# Supplementary material for: Integration of Bioinformatics Resources Reveals the Therapeutic Benefits of Gemcitabine and Cell Cycle Intervention in SMAD4-Deleted Pancreatic Ductal Adenocarcinoma
Source: Genes (Basel). 2019 Sep 28;10(10):766. doi: 10.3390/genes10100766 (PMC6827004; doi:10.3390/genes10100766)
Supplement: Supplementary file 1 [file genes-10-00766-s001.zip › Table S6.docx]

**Table S6. Genes correlated with gemcitabine sensitivity in PDAC patient-derived organoids.** *Data source: Cancer Discovery. 2018;8:1112-1129.*

| **Gemcitabine-sensitive genes** | | **Gemcitabine-resistant genes** | |
| --- | --- | --- | --- |
| ASNSD1  CISD3  TBCD  CASKIN2  ARHGDIA  PNPO  DUSP14  CENPM  TACO1  GUF1  GOT2  CUTC  MSH6  KHK  STARD3  FAM102B  POLR2D  LRRC45  MMD  COA3  RNF43  KIF18B  RHOBTB3  BIN1  GIT1  MRTO4  HIRA  ACACA  PISD  RANBP1  SRRT  TMEM177  CNP  CNOT9  DPY19L1  CTU2  AP2B1  PRMT6  TRMU  NCAPH2  UBE2L3  ANKRD40  SLC25A39  CCAR1  MICAL3  MRPL27  SELENOI  KPNB1  DNAJC2  DUS1L  NT5C3B  SLFN11  RCC1  METAP1  MIIP  SPECC1L  CUL1  MAIP1  ABCE1  ILKAP  FGFR1OP  IMP4  NF2  HNRNPD  COPRS | PAQR3  TRRAP  SNRNP40  HSPE1  POM121C  ZNF398  HCFC1  GPAM  ATAD5  CENPX  PAICS  WASF1  HTT  BIRC5  RFC1  MCM6  PPIG  RIF1  PDS5A  RBM28  UBA6  CPSF4  PAXIP1  RNF166  MTFP1  TOMM22  PRIMPOL  ESS2  SLBP  ZC3H15  LSM6  ZDHHC16  NAA15  CCDC117  RPS6KA6  WDR33  TSN  MRPL12  DTYMK  SLC25A10  SMTN  RFC2  TAF6  RAD51C  GINS2  PSMC3IP  AADAT  CEP131  CEP135  METTL21A  SSB  UBXN2A  ABCB6  GNA12  FLCN  LETM1  RANGAP1  RCC1L  ENOPH1  PDAP1  SMPD4  ZNF511  NSD2  MRFAP1  FAM133B | CREB3L4  TMC4  SENP7  BICDL2  MARF1  CALCOCO1  OS9  TPD52  DNAH1  TMEM254  NRBP2  CSAD  MEF2D  ESRP1  SLC35E1  KDM5B  HBP1  SMPD3  SIM2  SLC50A1  TCEA3  CEBPD  KLHL24  PCED1A  INTS3  LIPH  SLC27A1  TSPAN31  RNF19A  AP3S1  SDC4  KLHL3  BRWD1  GOLPH3L  FXYD3  BCL6  YPEL5  S100A13  ELL  TSTD1  LETMD1  DUSP4  ALDH1L2  MIA3  PCMTD2  R3HDM4  TMEM163  RALGPS1 | SLU7  CAB39L  SDHC  ATF6  TMEM59  NCSTN  PBXIP1  PNMA2  ERLEC1  STXBP2  ACAD10  ELMO3  KRTCAP2  TFF3  ADGRE5  MMP28  SIDT2  ZFP64  RAB25  COPA  SMIM19  KDM3A  LYST  PEX11B  RSPH1  SPIRE2  FP565260.3  PAN2  RRNAD1  MAGI1  FBXO46  DCAF8  SPPL2B  NBPF12  C20orf194  PPM1L  ERN2  HSF4  SEZ6L2  ITM2B  N4BP2L2  MC1R  THBS3  ZFC3H1  ZDHHC11  GTF2IRD2B  LMF1 |
