# Supplementary figures and images for: Integration of Bioinformatics Resources Reveals the Therapeutic Benefits of Gemcitabine and Cell Cycle Intervention in SMAD4-Deleted Pancreatic Ductal Adenocarcinoma
Source: Genes (Basel). 2019 Sep 28;10(10):766. doi: 10.3390/genes10100766 (PMC6827004; doi:10.3390/genes10100766)

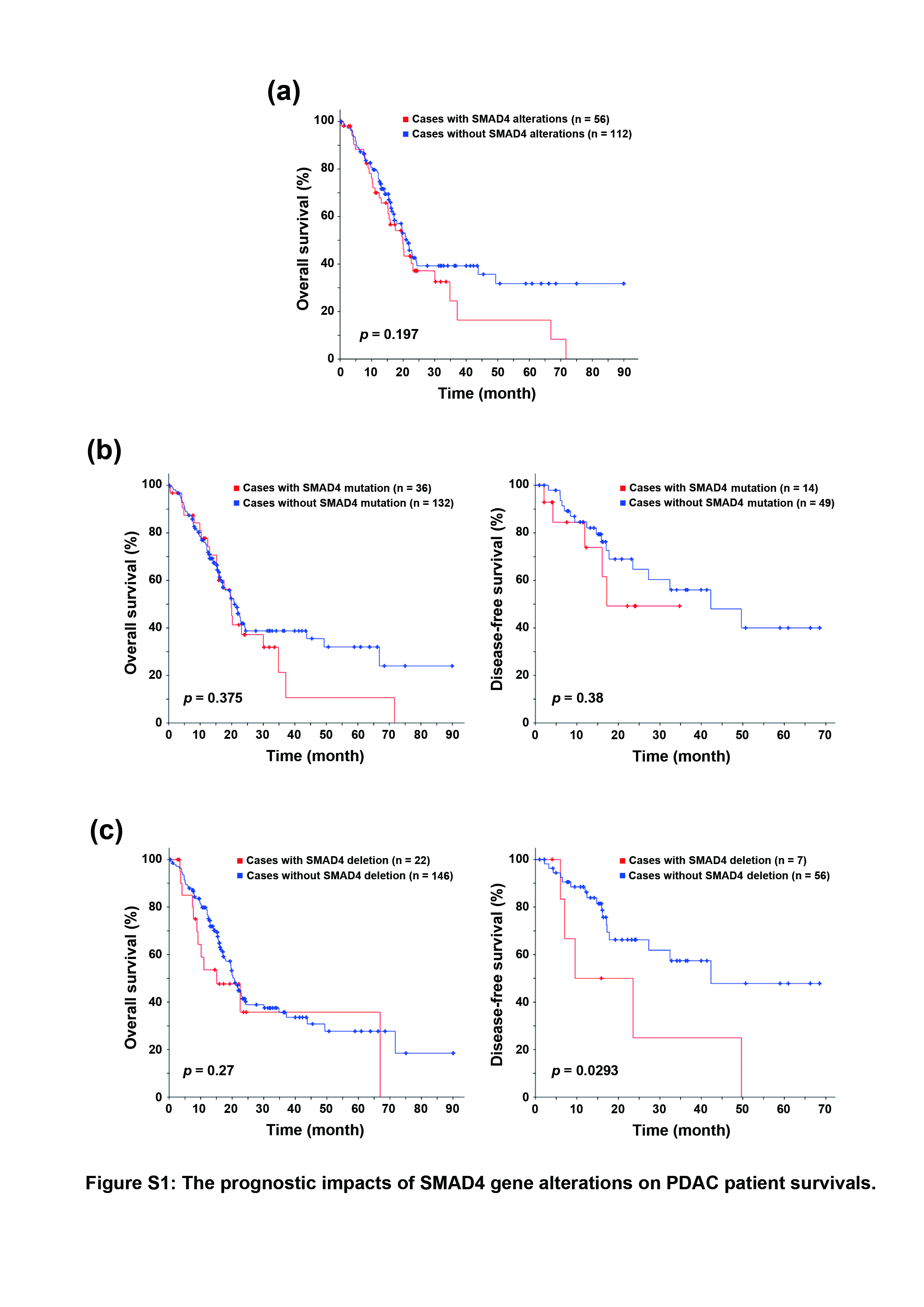

Supplement: Supplementary file 1 [file genes-10-00766-s001.zip › Figure-S1.tif]

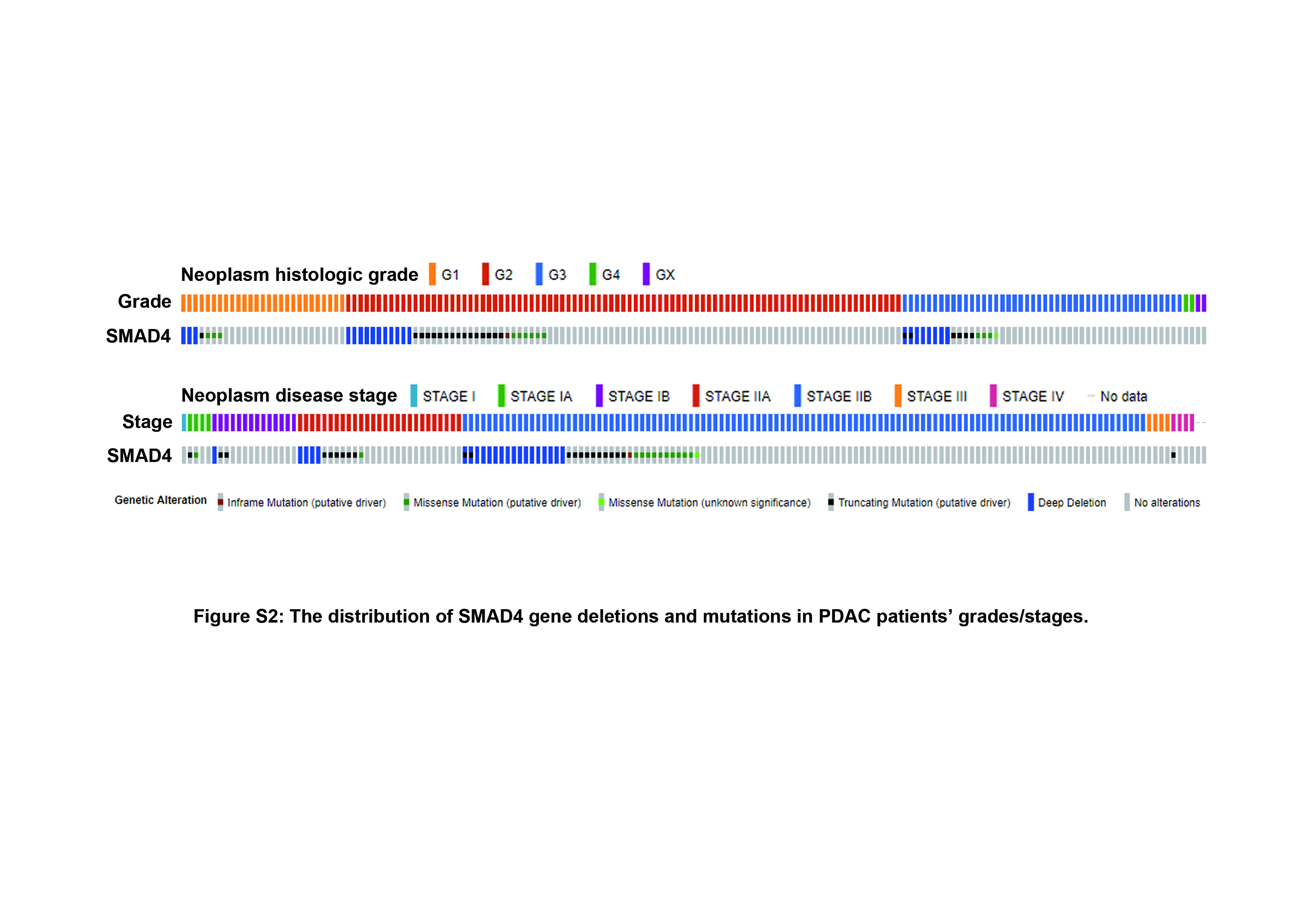

Supplement: Supplementary file 1 [file genes-10-00766-s001.zip › Figure-S2.tif]

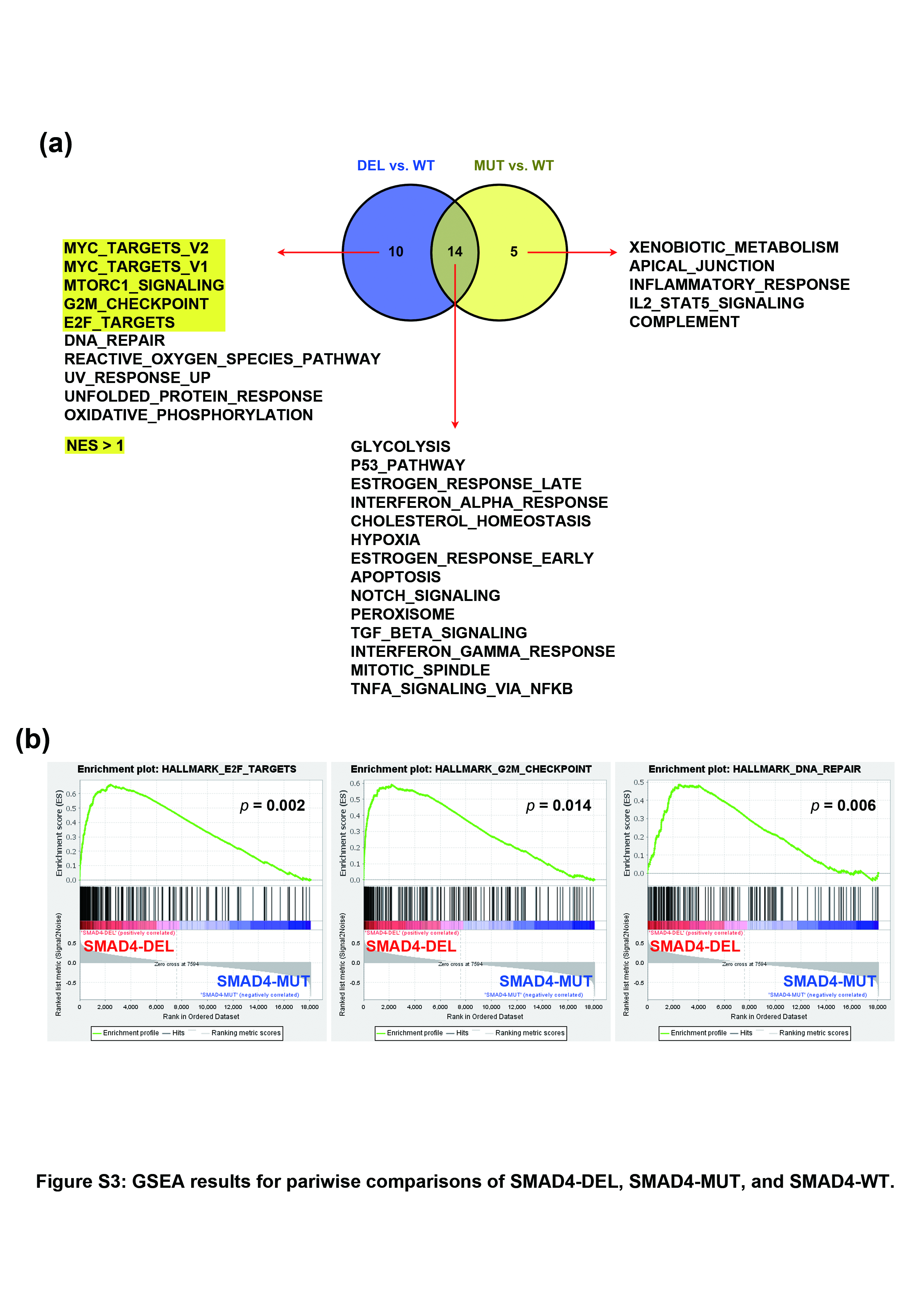

Supplement: Supplementary file 1 [file genes-10-00766-s001.zip › Figure-S3.tif]

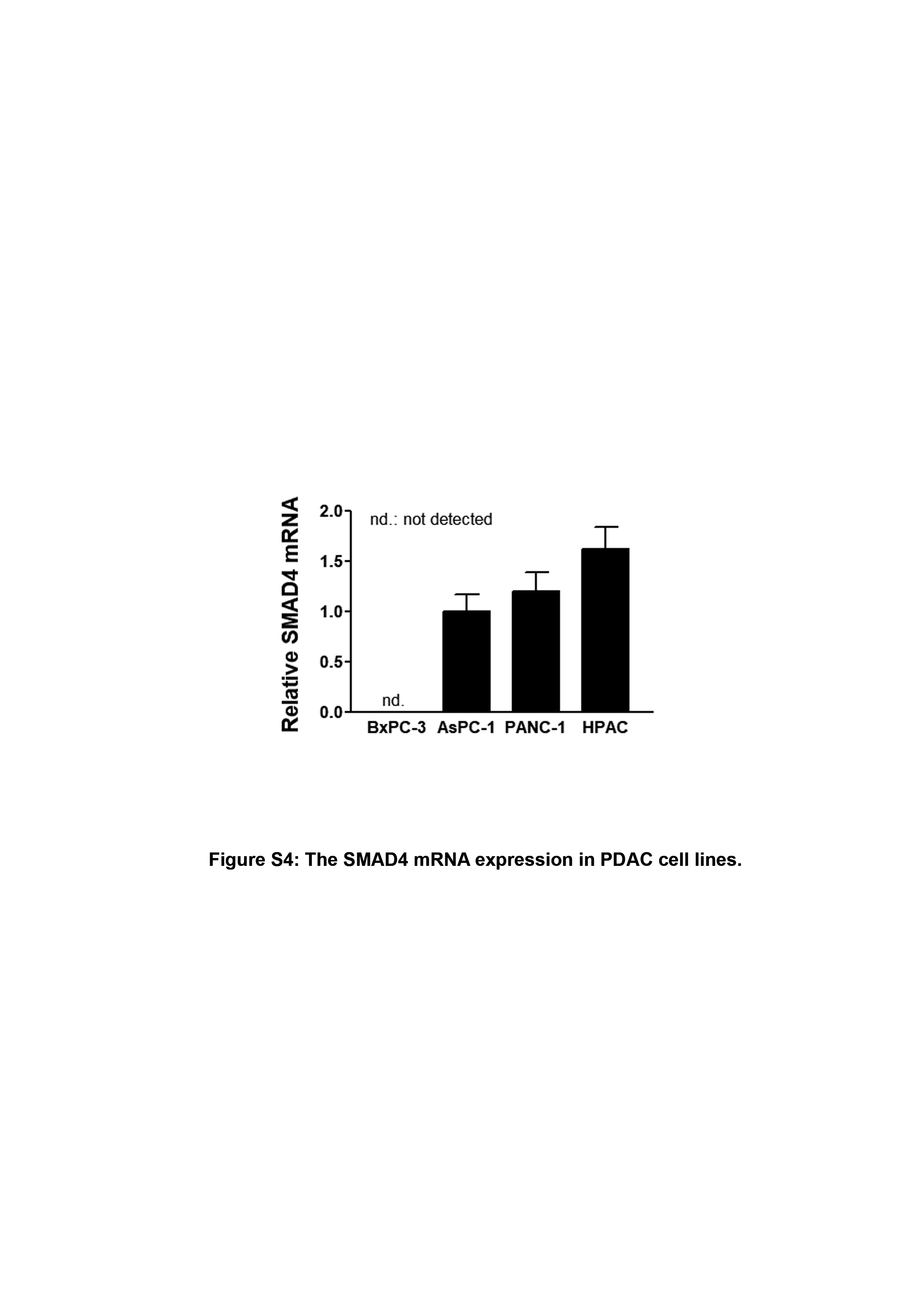

Supplement: Supplementary file 1 [file genes-10-00766-s001.zip › Figure-S4.tif]

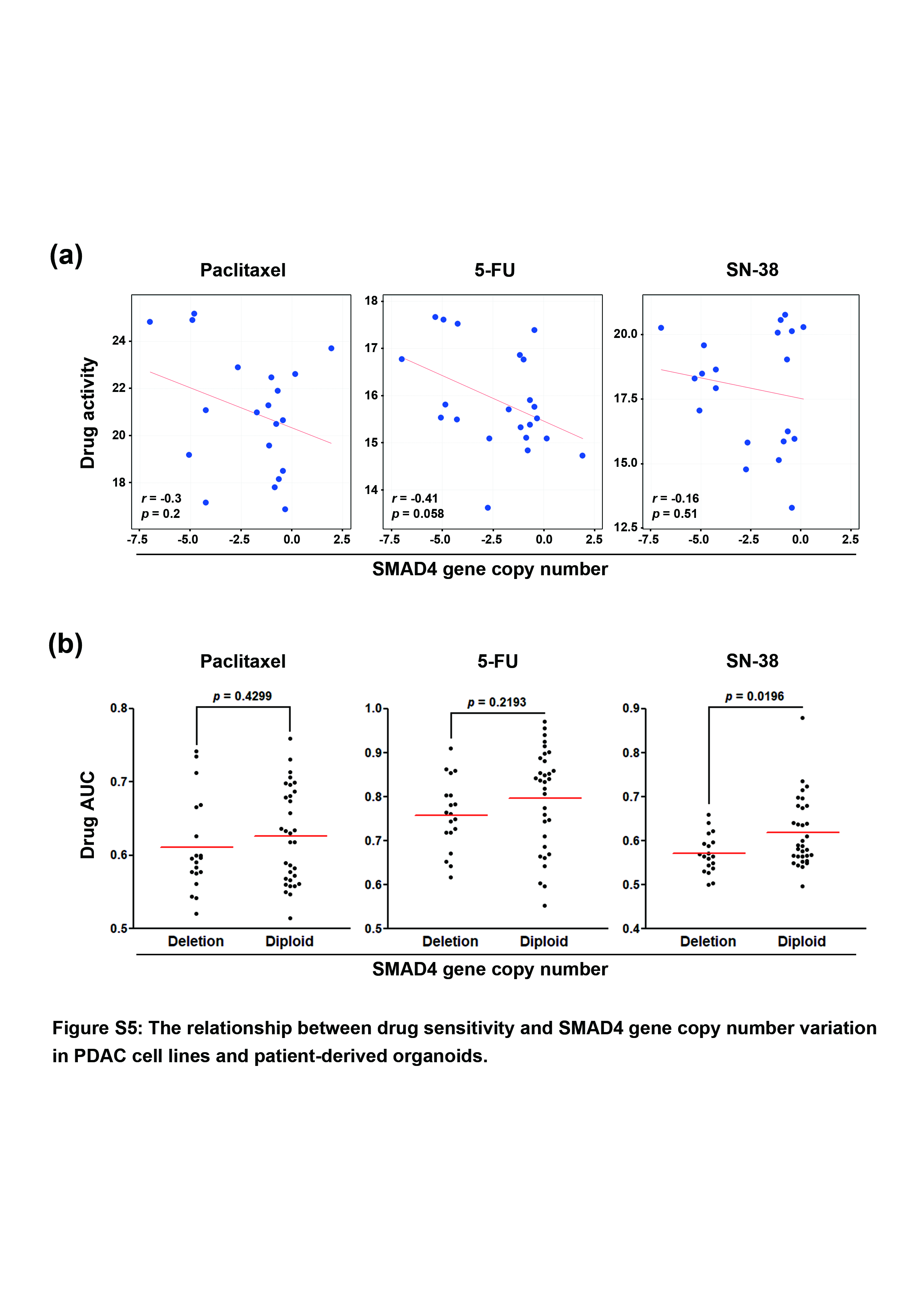

Supplement: Supplementary file 1 [file genes-10-00766-s001.zip › Figure-S5.tif]

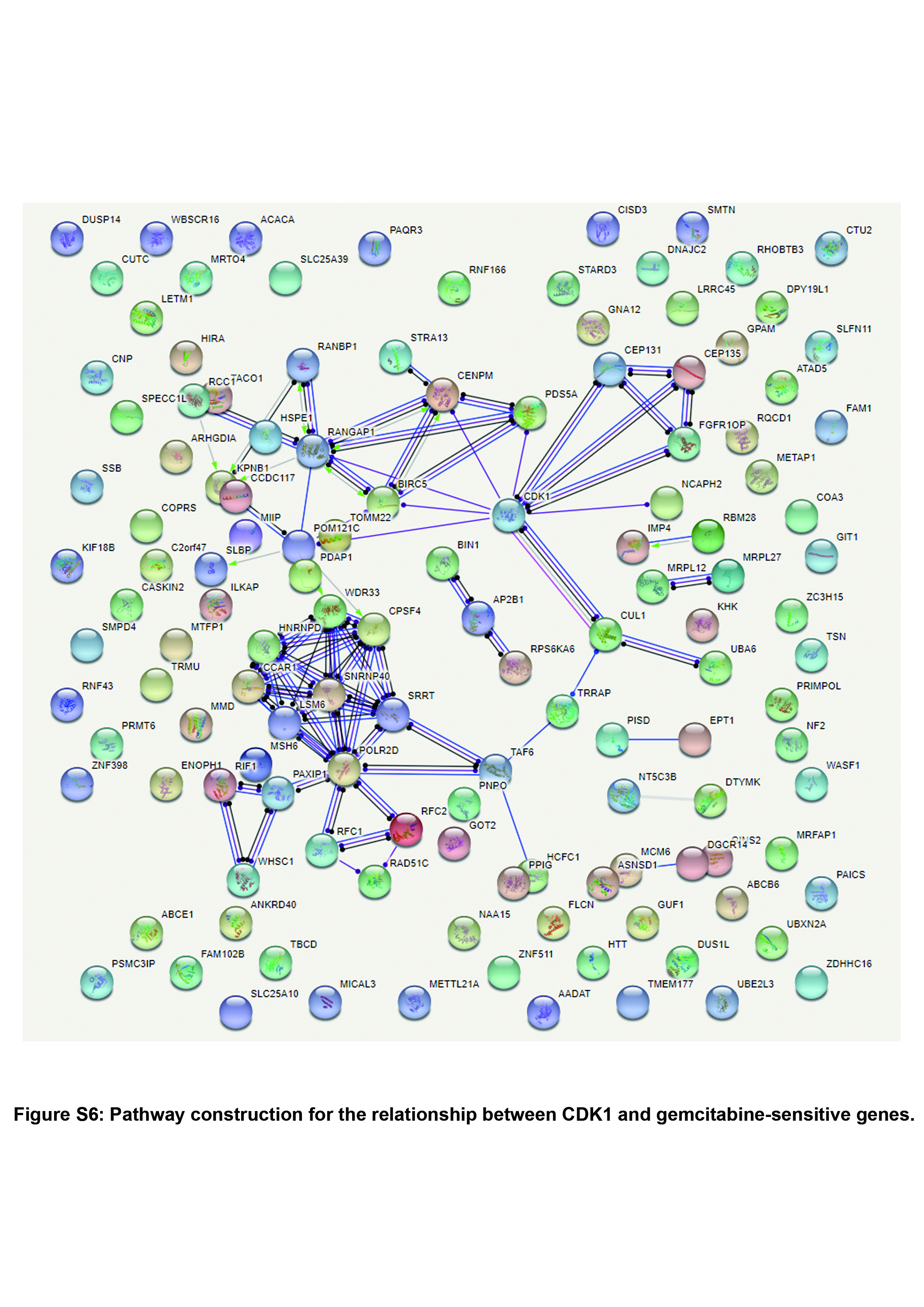

Supplement: Supplementary file 1 [file genes-10-00766-s001.zip › Figure-S6.tif]

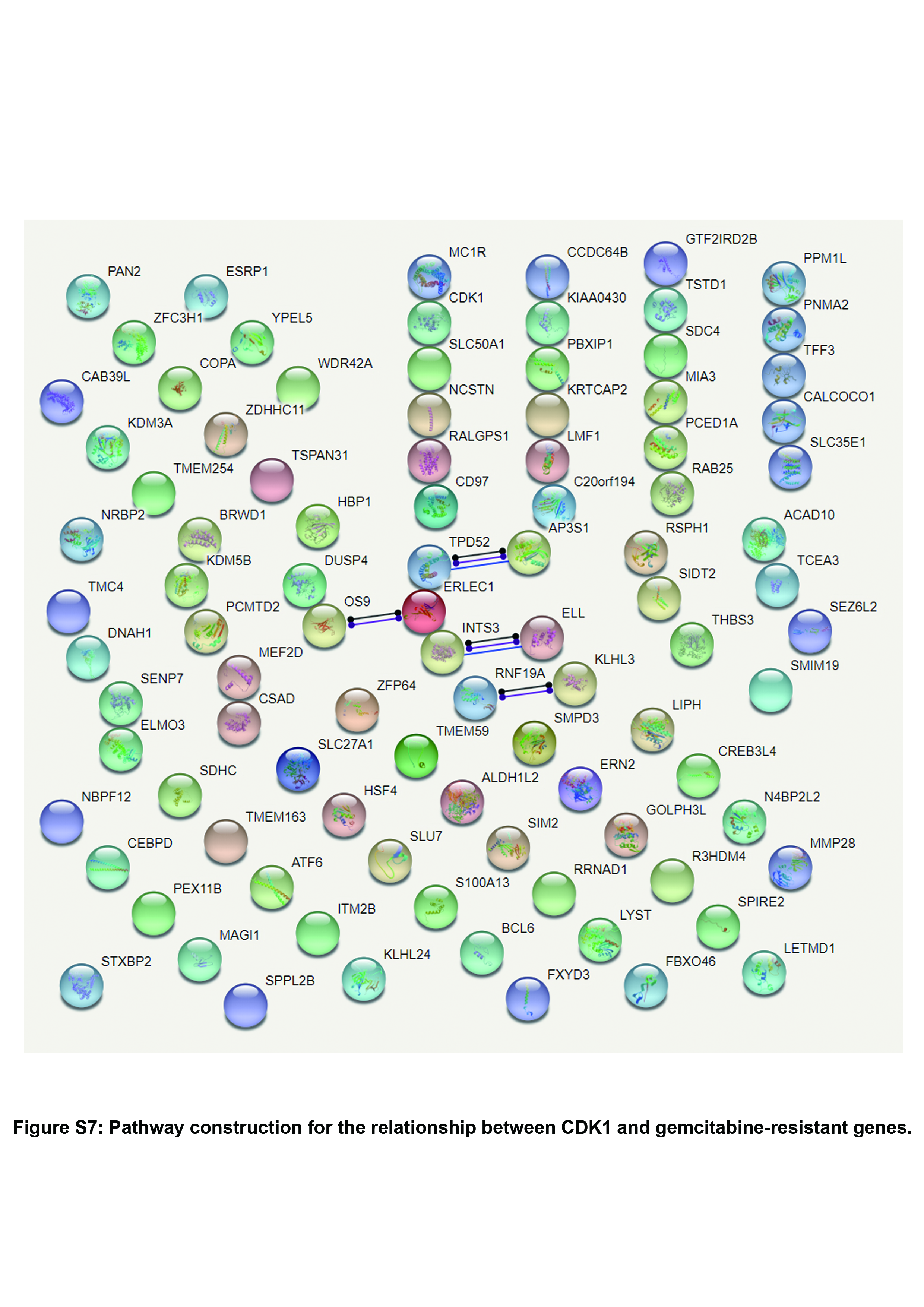

Supplement: Supplementary file 1 [file genes-10-00766-s001.zip › Figure-S7.tif]

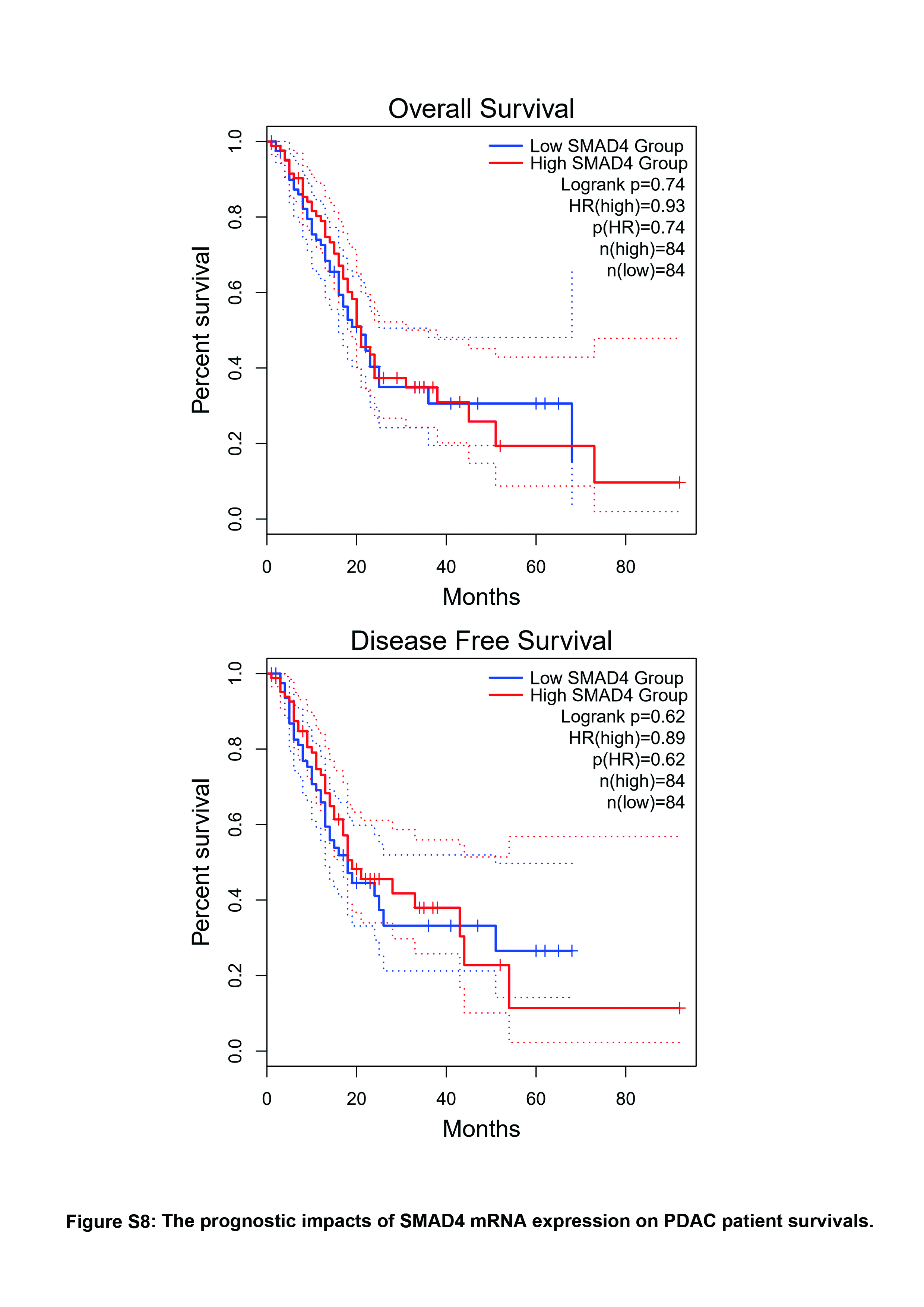

Supplement: Supplementary file 1 [file genes-10-00766-s001.zip › Figure-S8.tif]
